# Evaluation of Anti-Inflammatory, Anti-Platelet and Anti-Oxidant Activity of Wine Extracts Prepared from Ten Different Grape Varieties

**DOI:** 10.3390/molecules25215054

**Published:** 2020-10-30

**Authors:** Elizabeth. Fragopoulou, Filio Petsini, Maria Choleva, Maria Detopoulou, Olga S. Arvaniti, Eftyhia Kallinikou, Eleni Sakantani, Ageliki Tsolou, Tzortzis Nomikos, Yiannis Samaras

**Affiliations:** 1 Department of Nutritions & Dietetics, School of Health Sciences & Education, Harokopio University, 17676 Athens, Greece; petsini.filio@gmail.com (F.P.); mcholeva@hua.gr (M.C.); mdetopoulou@gmail.com (M.D.); tnomikos@hua.gr (T.N.); 2 Department of Food Science and Technology, Faculty of Environmental Sciences, Ionian University, 17676 Athens, Greece; olga.arvan@gmail.com (O.S.A.); efikal6@yahoo.gr (E.K.); esakadani@ionio.gr (E.S.); atsolou@ionio.gr (A.T.); ysamaras@ionio.gr (Y.S.)

**Keywords:** oxidative stress, inflammation, platelet aggregation, wine extract, biological score, platelet activating factor, thrombosis

## Abstract

Inflammation, thrombosis and oxidative stress are rarely studied together when wine’s biological activity is concerned; hence the existing literature lacks a holistic point of view in the biological outcome. The scope of the present study is to parallel evaluate the effect of wine extracts on those mechanisms. Ten wine varieties and two different extraction methods were used leading to five extracts for each wine: total lipids (TL) and fractions with different phenolic compound classes (FI, FII, FIII and FIV). Their effect on oxidative stress, platelet aggregation and the secretion of cytokines from mononuclear cells was measured and a biological score was calculated. FII of white wines is the most potent extract and the extracts FIII and TL are following. Specifically, FII had higher anti-oxidant and anti-inflammatory score while all three fractions had a similar anti-platelet score. Furthermore, FII and FIII extracts were the most potent red wine extracts and revealed the highest anti-oxidant and anti-inflammatory scores. White wine FII extracts were more potent than the red wine ones while FI and FIV extracts of red wine were more potent than the white wine ones. In conclusion, the protective effect of a wine is independent of its color but is strongly associated with its microconstituents profile. FII extract revealed the highest biological score and further examination is needed in order to identify the compounds that are responsible for the aforementioned actions.

## 1. Introduction

Several experimental and epidemiological studies have demonstrated a favorable effect of light to moderate wine consumption on the cardiovascular system [1,2]. The underlying mechanisms of atherosclerosis, the main cause of cardiovascular diseases, are oxidative stress, inflammation [3] and thrombosis [4]. The above mechanisms interact in both the onset and the progression of the disease. Atherosclerosis is initiated when endothelial dysfunction along with platelet activation are induced by disturbed shear stress or imbalance on inflammatory and oxidative stress status that can lead to endothelial dysfunction and platelet activation. Subsequently, monocytes adhering on the activated endothelium, migrate in the subendothelium and differentiate into proinflammatory macrophages, which increase the uptake of oxidized low density lipoprotein (oxLDL) leading monocytes to turn into foam cells, exacerbating the inflammatory signaling [5]. The platelet-activating factor (PAF) is an inflammatory and thrombotic mediator that is produced during LDL oxidation along with oxidized phospholipids, which are responsible for oxLDL’s proatherogenic effects [5,6]. Among other actions, PAF leads to endothelial dysfunction and platelet aggregation, while it is involved in monocytes migration into the subendothelium and foam cell formation [6].

The cardio protective effects of wine consumption versus other alcoholic beverages are thought to be attributed to its microconstituents. These compounds could be either water or lipid soluble and therefore could reveal several biological actions in different target sites of the human body. Among them, the presence of numerous phenolic compounds has been reported, including compounds such as flavanols (e.g., cetechins), flavonols (e.g., quercetin), stilbenes (e.g., resveratrol), phenolic acids (e.g., gallic acid) and phospho- and glyco-lipids [7,8]. Plethora data support the idea that wine microconstituents could exert anti-oxidant, anti-inflammatory and anti-platelet activities. In this line, our previous data revealed that wine extracts and/or individual wine phenolics could inhibit PAF-induced washed rabbit platelet aggregation [9,10,11], inhibit lipoxygenase activity and scavenge free radicals [12] and reduce PAF biosynthesis in U937 monocytes under basal and inflammatory conditions [13,14]. However, even though our previous results indicate the importance of the variety over the color regarding the observed biological activity, no in depth analysis was performed in order to evaluate different wine varieties. Moreover, the three pathological mechanisms are rarely studied together; hence the existing literature lacks a holistic point of view in the biological outcome.

Therefore, the purpose of the present study is to assess in parallel the effect of ten indigenous wine varieties on the three pathological mechanisms and to evaluate their biological activity with a holistic biological score. In particular, in order to evaluate the biological activity, radical scavenging capacity, inhibitions of metals- or enzyme- induce oxidative stress, inhibition of human platelet aggregation against four agonists and the secretion of cytokines from peripheral blood mononuclear cells (PBMCs) were measured.

## 2. Results

Five extracts (TL, FI, FII, FII and FIV) from ten wines, five white and five red, were tested for their biological activity against oxidative stress, thrombosis and inflammation. For simplicity reasons the results are presented with regard to the color of the wine and the type of the extract (Table 1, Table 2, Table 3 and Table 4). The corresponding data for each wine separately is presented in the supplementary tables (Tables S1–S4).

### 2.1. Chemical Determinations

The extract with the higher total phenolic concentration was the FII (*p_s_* < 0.000) followed by the extract FIII (*p_s_* < 0.05) while the extracts TL, FI and FIV exhibited similar but lower total phenolic concentration. The comparison between red and white wines showed that FI extracts of red wines contained higher total phenolic compounds compared to white wines (*p* = 0.01) while regarding the other extracts no significant differences were observed (Table 1). Ortho-phenolic compounds revealed higher concentration in extracts FII and FIII compared to extracts TL, FI and FIV (*p_s_* < 0.000). The only difference observed between red and white wines was the higher concentration of ortho-phenolics in extract FIII of white wines compared to the red ones (*p* = 0.04; Table 1). As far as the phosphorus content is concerned, FI extracts had higher concentration in comparison to all the other extracts (*p_s_* < 0.000). FII extracts of white wines had lower phosphorus concentration versus the ones of red wines (*p* = 0.02; Table 1). Finally, sugar determination revealed that FI extracts comprised of significantly higher sugar amounts than FIII extracts (*p* = 0.04) but no other significant difference was observed. In addition, extracts FII (*p* = 0.002) and FIII (*p* < 0.001) contained less sugars in white wines compared to red ones (Table 1). It should be noted that Mavrodaphne’s (red wine) FIV extract revealed much higher concentration of both phenolic compounds and sugars than any other wine’s FIV extract (Supplementary Table S1).

### 2.2. Anti-Oxidant Activity

Regarding the scavenging activity of DPPH radicals, extracts TL (*p_s_* < 0.05), FII (*p_s_* < 0.01) and FIII (*p_s_* < 0.005) revealed similar capacity, always greater than the capacity of FI and FIV extracts. In Table 2, the anti-oxidant properties of the extracts are presented separately for white and red wines. The comparison between white and red wines showed that FII (*p* < 0.05) extracts of red wines had lower capacity (higher IC_50_ value) to scavenge free radicals than white ones, while the opposite was observed for FI extracts (*p* < 0.001). The ability of extracts to inhibit LOX activity revealed that extracts TL (*p* = 0.002), FII (*p* < 0.000) and FIII (*p* < 0.000) had similar capacity but also higher when compared to the FI fraction. It should be noticed that FIV extracts revealed no inhibitory activity against LOX. The comparison between white and red wines showed that FII (*p* = 0.01) extracts of red wines had lower capacity (higher _IC50_ value) to inhibit LOX than white ones while the opposite was observed for FI extracts (*p* < 0.001). On the other hand, all the extracts managed to inhibit Fe-induced oxidation of LA but no significant difference was observed either among the extract type or between red and white wines with respect to each extract. Finally, extracts FII (*p_s_* < 0.001) presented the highest ability to inhibit Cu-induced serum oxidation among all other extracts. Meanwhile, red wine extracts FI (*p* = 0.005), FII (*p* = 0.003) and FIV (*p* = 0.05) displayed greater capacity towards serum oxidation inhibition versus the white wine ones.

### 2.3. Anti-Platelet Activity

The anti-platelet activity of extracts was tested against five agonists namely PAF, ADP, TRAP, collagen and arachidonic acid (AA). All extracts revealed similar anti-platelet activity against PAF and TRAP (*p_s_* > 0.05). When the analysis was performed with regard to the color of the wine, it was observed that white wine extracts TL, FII and FIII were more potent inhibitors of PAF-induced platelet aggregation (Table 3). TL, FII and FIII fractions inhibited more potent platelet aggregation against ADP and collagen (lower IC_50_ values) compared to fractions FI and FIV (*p_s_* < 0.05). When the analysis was performed with regard to the color of the wine it was noticed that white wine extracts TL, FII and FIII were more potent inhibitors of ADP-induced platelet aggregation (Table 3). Anti-platelet activity against AA revealed that extracts TL, FII and FIII were more potent inhibitors than FI and FIV (*p_s_* < 0.01) and this result was more pronounced in white wines as it is shown on Table 3. The comparison between red and white wines demonstrated that red wine FI extracts were more potent inhibitors of platelet aggregation compared to white wines regardless of the agonist. Additionally, FII and FIV extracts of red wines were more potent inhibitors against TRAP-induced platelet aggregation.

### 2.4. Anti-Inflammatory Activity

The ability of extracts to reduce LPS-induced TNF-α and Il-1β secretion was tested in PBMCs from healthy volunteers (Table 4). Initially, several amounts of extracts were used in order to find the optimum concentration for each extract. In general, 500 μg of TL, FII and FIII extracts decreased cytokine secretion. In particular, FII extracts were more potent than TL and FIII extracts (*p_s_* < 0.005) and this result was more pronounced in white wines. As far as the extracts FI and FIV are concerned, they expressed similar inhibitory activity at 100 μg against LPS-induced cytokine secretion (TNF-α *p* = 0.73, IL1b *p* = 0.95) in red wines whereas in the case of white wines, the FIV fraction was more potent an inhibitor compared to FI (*p* = 0.02). It should be mentioned/acknowledged that at 500 μg the FI and FIV extracts caused a slight activation of LPS action. The comparison between red and white wines showed that white wine FII extracts inhibited LPS-induced Il-1β secretion more effectively than red wine ones (*p* = 0.05) while a similar trend was observed in the case of TNF-α secretion (*p* = 0.08). No significant difference was observed in FI and FIV extracts between red and white wines.

### 2.5. Biological Score

**Figure 1:**
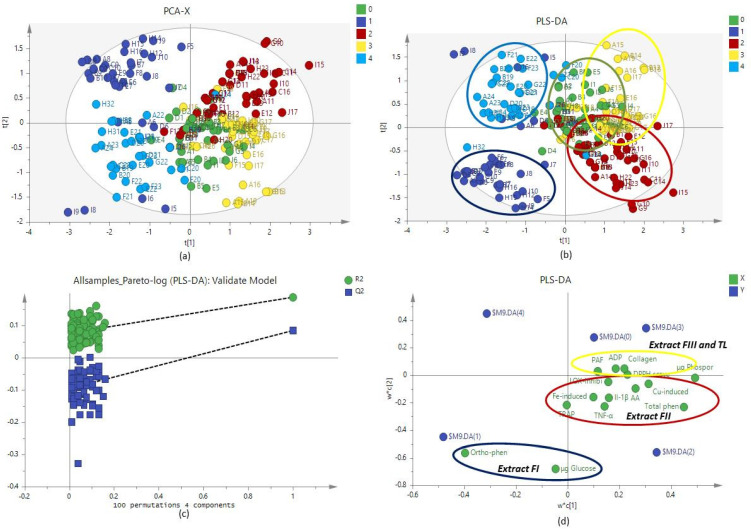
Graphs of the multivariate analysis: (**a**) PCA scores plot of fractions; (**b**) PLS-DA scores plot; (**c**) permutation test with 100 permutations for the PLS-DA model and (**d**) loading plot. 0: TL extract; 1: FI extract; 2: FII extract; 3: FIII extract; 4: FIV extract.

In order to obtain a holistic point of view of the biological activity of the extracts a biological score, based on each mechanism, was created and presented on Table 5. Extracts FII seemed to be the most potent ones followed by TL and FI. Additionally, white wine FII fractions revealed a better biological activity than the red wine ones. Finally, in all extracts the anti-oxidant biological score was positively correlated with total phenolic content (r = 0.641, *p* < 0.000), ortho-phenolic content (r = 0.624, *p* < 0.000) and sugars (r = 0.756, *p* < 0.000), the anti-platelet score positively correlated with phosphorus (r = 0.387, *p* < 0.01) and (r = 0.400, *p* = 0.009) sugar content while no significant correlation was observed for the anti-inflammatory score. The significant correlations between biological scores and chemical determination are presented in Table 6. It is notable that the biological activity of the FII fraction is mainly correlated to ortho-phenolic compounds while the biological activity of the TL extract correlated to phosphorus and the sugars content. The data were subjected to statistical multivariate analysis. The unsupervised analysis (Principal component analysis, PCA) is presented in Figure 1a with satisfactory descriptive and predictive capability (R2: 0.686/Q2: 0.373). Three groups of classification are presented: FI and FIV fractions (with the lower biological activity), FII fractions (with the highest biological activity) and FIII and TL fractions (with also potent biological activity but lower than FII fractions). The application of supervised sample classification using partial least square discriminant analysis, (PLS-DA) did not reveal a different classification pattern but mainly improved the existing intrinsic clustering, already described by the PCA model (Figure 1b). The score plots of the generated PLS-DA model is shown in Figure 1d. The classification of the FII fraction was attributed to anti-oxidant activity, inhibition of TNFa and IL1β secretion and anti-platelet action against AA and TRAP while the classification of FIII and TL extracts was attributed to anti-platelet action against PAF, ADP and collagen (Figure 1d).

Finally, a preliminary detection of some phenolic compounds was performed on the FII extract, which revealed the greater biological score in one white (Robola of Kefalonia) and one red (Vertzami) wine (Supplementary Figure S1). The results indicated that the concentration of gallic acid was 347.3 ± 4.0 μM and 14.5 ± 1.1 μM, of tyrosol 3467.6 ± 539.9 μM and 4693.1 ± 457.3 μM, of catechin 119.9 ± 21.4 and 54.2 ± 2.9, of chlorogenic acid 470.5 ± 4.1 μM and 987.1 ± 10.8 μM; of resveratrol 18.9 ± 0.5 μM and 36.1 ± 1.0 μM and of quercetin 7.05 ± 0.50 μM and 1.49 ± 0.13 μM, respectively.

## 3. Discussion

Atherosclerosis is the common pathological mechanism behind the cardiovascular diseases. It is characterized as a multifactorial pathological situation in which the interactions among oxidative stress, thrombosis and inflammation are crucial for the outcome of the diseases. Epidemiological data support the J-shaped relationship between wine consumption and vascular events and cardiovascular mortality [2]. In the present study we performed, for the first time, the evaluation of the biological activity of ten different wine varieties against oxidative stress, thrombosis and inflammation concurrently. The results indicate a specific extract as the most potent fraction that possesses anti-oxidant properties could inhibit the platelet aggregation against five agonists and the LPS-induced Il-1β and TNF-α secretion could form PBMCs.

The beneficial effect of wine versus other alcoholic beverages is mainly attributed to its microconstituents. During the last decades, an effort was made to isolate and identify the biological active compounds that are responsible for the wine’s cardio protective effect [7]. Wine consists of a complex mixture of compounds that originate both from the grapes and the fermentation process. It is worth mentioning that red wine fermentation is performed in the presence of the grape skins while in white wine it is performed in their absence. This difference results in approximately tenfold higher phenolic compounds content in red wines versus white ones. The above fact led researchers to the initial opinion that red wines have stronger cardioprotective properties than white wines. Currently, several reports support the idea that some white wines can provide cardio protection similar to red wines [10,15,16]. However, the effect of wine color to its biological activity is still a debate and for this reason red and white wines were both included in the present study.

The protective effect of bioactive compounds in wine was initially attributed to their anti-oxidant properties, but currently it is generally accepted that they exert potent anti-thrombotic and anti-inflammatory actions [1,17,18]. In this line, our previous data revealed that wine extracts exert potent anti-platelet and anti-inflammatory activity. More specifically, it was shown that wine extract inhibits PAF-induced platelet aggregation [9,10,11], the biosynthetic enzymes responsible for PAF biosynthesis [13,14] and also lipoxygenase activity [12]. PAF is a potent inflammatory and thrombotic mediator that participates in the initiation and prolongation of atherosclerosis [5,6]. Its inhibition from wine microconstituents could partly explain the cardio protective effect of wine consumption [7].

In order to evaluate the influence of the color and the variety of grape in the observed biological activity, five red wine varieties, namely Petrokoritho, Vertzami, Avgoustiatis, Red thiako and Mavrodaphne of Kefalonia, and five white wine varieties, namely Robola of Kefalonia, Tsaousi, Kakotrigis, Muscat of Kefalonia and White thiako, were chosen. Two extraction methods were performed in each wine; the first was performed in order to obtain wines’ total lipid fractions and the second in order to obtain fractions with different polyphenolic content since phenolics constitute the majority of bioactive compounds in wines [12]. Therefore, in the second extraction method four different fractions were obtained; fraction FI contains all the anthocyanic compounds, FII, the subclass of procyanidins, catechins and several flavonols (mainly quercetin-3-O-glucoside), whereas FIII contains phenolic acids and quercetin-3-O-glucuronide. Fraction FIV contains the rest of the components that are not distributed to previous fractions [19]. In each extract 11 biological assays were performed, in cell free systems and cell culture systems, against oxidative stress, thrombosis and inflammation, in order to achieve a holistic point of view of their biological activity.

Oxidative stress is complex situation where many aspects must be taken into account so as one to address it. Therefore, in order to evaluate the anti-oxidant capacity of the extracts, four different assays were performed. The antiradical activity of extracts was observed using the DPPH free radical assay, while their ability to inhibit the enzymatic fatty acid’s peroxidation was determined through inhibition of lipoxygenase activity. Their ability to inhibit the non-enzymatic fatty acid’s peroxidation was obtained through inhibition of Fe-induced fatty acid oxidation and, finally, their total antioxidant activity was determined via inhibition of Cu-induced serum oxidation. The results indicated that the extracts TL, FII and FIII revealed a similar capacity regarding scavenging DPPH radicals and inhibiting LOX activity, always greater when compared to extracts FI and FIV. Additionally, FII fraction revealed a higher ability to inhibit Cu-induced serum oxidation than any other fraction. On the contrary, no significant difference was observed among the extracts when inhibiting Fe-induced oxidation. The comparison between white and red wines showed that the FII extracts of the red wines were more potent to scavenge free radicals and to inhibit LOX than the ones of white wines, though the opposite was observed for FI extracts. Additionally, red wine extracts FI, FII and FIV had a higher capacity to inhibit Cu-induced serum oxidation versus the white wine ones. In summary, as far as anti-oxidant capacity is concerned, FII extracts were the more potent ones, followed by TL and FI extracts while red wines seem to exert greater anti-oxidant activity than the white ones.

The inhibition of platelet aggregation and thrombus formation constitutes key steps in the prevention of cardiovascular events. Several studies reported that wine phenolic extract, dealcoholized red wine and its catechin-anthocyanidin fractions exert a significant effect on ADP-induced platelet aggregation [20,21,22]. Additionally, wine lipoid extracts from several grape varieties inhibited PAF-induced platelet aggregation. This effect was observed in both white and red varieties indicating that the grape variation was the main factor for the observed biological activity and not the color of the grapes. Indeed, a red wine (main variety cabernet sauvignon) and a white wine (main variety Rombola) appeared to be the most potent ones and apart from phenolic compounds as bioactive compounds, phospho- and gluco-lipids also exert anti-platelet activity [9,10,11]. Results from the corresponding musts revealed that the fermentation process in the red wines led to increased anti-platelet activity while in white wines the biological activity remained practically unchanged before and after fermentation. In order to have a better estimation of anti-platelet activity of the extracts, five agonists for platelet aggregation, namely PAF, ADP, TRAP, collagen and arachidonic acid, were used. These agonists act through different receptors and/or different signal pathways causing platelet activation and aggregation. PAF acts through its specific receptor (PAFR) and induces shape change, aggregation and release of granule contents [23]. ADP binds to two G-protein coupled receptors, P2Y1 and P2Y12, induces shape change and initiates primary wave platelet aggregation through calcium mobilization. Thrombin binds to protease-activated receptors 1 and 4. Collagen binds to the GpVI and GpIa/IIa receptors inducing granule release, thromboxane A2 (TXA2) generation and then prolonged GPIIb-IIIa activation. The GpIa/IIa receptor is involved in platelet adhesion while the GpVI receptor is involved in platelet signaling and TXA2 generation. Arachidonic acid is the precursor of TXA2 within platelets and is converted to TXA2 by cyclooxygenase and thromboxane synthase. Based on the results of the present study white wine extracts TL, FII and FIII were more potent inhibitors of platelet aggregation, than FI and FIV regardless of the agonist used. The comparison between red and white wines also revealed that red wine FI extracts were more potent inhibitors of platelet aggregation compared to white wines, regardless of the agonist used. Additionally, FII and FIV extracts of red wines demonstrated greater inhibition against TRAP-induced platelet aggregation compared to white ones. Wine microconstituents may exert their activity in platelet aggregation through modulation of platelet signal transduction such as by inhibiting protein kinase C [24] and phospholipase C [25] activities, or through PI3K/Akt inactivation, and cAMP elevation [26].

Several in vitro studies indicate that wine microconstituents could modulate inflammatory pathways through regulation of enzymatic activity and/or inhibition of the secretion and the expression of proinflammatory cytokines through modification of transcription factors. Wine extracts and resveratrol have been reported to inhibit the proinflammatory enzymes cyclooxygenase-1 [27], lipoxygenase [12] and also the enzymes of PAF biosynthesis, namely lyso-PAF-AT and PAF-CPT, under basal and inflammatory conditions [13,14], leading to the suppression of proinflammatory eicosanoids and PAF synthesis, respectively. In the present study, the ability of extracts to reduce LPS-induced TNF-α and Il-1β secretion was tested in PBMCs from healthy volunteers. FII extracts were the most potent ones compared to TL and FI extracts and this result was more pronounced in white wines. In the case of white wines the FIV fraction was more potent an inhibitor compared to the FI fraction. When compared, the white wine FII extracts were more potent inhibitors of LPS-induced Il-1β secretion compared to red wine ones while an analogous trend was observed in the case of TNF-α secretion. No significant difference was observed in FI and FIV extracts between red and white wines. Red wine extract has been reported to downregulate the expression of adhesion molecules at the mRNA and protein level [28]. Additionally, wine extracts exert a beneficial effect on intestinal inflammation and reduce IL-8 production and cyclooxygenase-2 expression [29], LPS-induced ICAM-1, VCAM-1 and PECAM-1 mRNA expression [30] and IL-1β secretion and gene expression in macrophages [31].

Since inflammation, oxidative stress and thrombosis interact inside the body and wine-microconstituents exert their effect against all of them, a biological score was created in an attempt to achieve a holistic estimation of the extracts’ biological activity. As far as white wines are concerned, FII is the most potent extract and extracts FIII and TL are following. Specifically, FII had higher anti-oxidant and anti-inflammatory score while all three fractions had similar anti-platelet score. Furthermore, FII and FIII extracts were the most potent ones in red wines and revealed higher anti-oxidant and anti-inflammatory scores compared to the other extracts. White wine FII extracts were more potent that the ones of red wines while the extracts FI and FIV of red wines were more potent than the ones of white wines. Finally, PCA analysis revealed that the classification of FII is based mainly to the inhibition of cytokine secretion, anti-oxidant actions and anti-platelet action against AA and TRAP while the classification of TL and FIII extracts were based on anti-platelet action against PAF, ADP and collagen.

In conclusion, the above results support the idea that both white and red wines contain biological potent microconstituents against oxidative stress, inflammation and thrombosis. Therefore, the protective effect of a wine is independent of its color but is strongly associated with its microconstituents profile. FII extract is the most potent one and further examination is needed in order to identify the compounds that are responsible for the aforementioned actions.

## 4. Materials and Methods

### 4.1. Reagents and Chemicals

Sodium molybdate dihydrate, perchloric acid 70–72%, Folin–Ciocalteau reagent and organic solvents were purchased from Merck (Darmstadt, Germany). Phenolic compounds, 2,2-diphenyl-1-picryhydrazyl (DPPH), soybean lipoxygenase (type I-B), linoleic acid and all other chemicals were purchased from Sigma-Aldrich (St.Louis, MO). All reagents and chemicals used were of analytical grade. RPMI 1640 and New Born Calf Serum (NCS) were purchased from BIOSERA (Nuaille, France) and LymphoprepTM from STEMCELL technologies (Vancouver, Canada). Bovine Serum Albumin (BSA) was obtained from Tocris Bioscience (Bristol, UK) and Human IL-1 beta/IL-1F2 and Human TNF-alpha DuoSet ELISA from R&D Systems (Minneapolis, MN, USA). L-glutamine, penicillin-streptomycin and all other reagents and solvents were supplied from Sigma (St.Louis, MO, USA).

### 4.2. Preparation of Wine Extracts

Ten wines from 5 white varieties (Robola of Kefalonia, Tsaousi, Kakotrigis, Muscat of Kefalonia and White thiako) and 5 red varieties (Petrokoritho, Vertzami, Avgoustiatis, Red thiako and Mavrodaphne of Kefalonia) were chosen. Two different extraction techniques were performed in order to collect either the total lipid fraction or several fractions containing different classes of phenolic compounds.

### 4.3. Extraction of Wine Total Lipids by the Bligh–Dyer Method

Total lipids (TLs) were extracted from the wine in accordance with the method of Bligh and Dyer [32], taking into consideration the ethanol content of each wine. The chloroform phase that contains the total lipids (TLs) was evaporated and dissolved in ethanol. All fractions were sealed under nitrogen and stored at −20 °C for further analysis.

### 4.4. Extraction of Wine in Order to Obtain Different Classes of Phenolic Compounds

Liquid/liquid extraction methods were performed in order to obtain several fractions containing different classes of polyphenolic compounds as previously described [12]. Briefly, the ethanol content was removed by vacuum distillation (at 30 °C and 30 mbar). A 150 mL aliquot of the dealcoholized wine (pH 2.0) was first extracted with ethyl acetate (three times with 100 mL of ethyl acetate each), obtaining an aqueous residue (FI fraction) and an organic phase. The organic phase (ethyl acetate) was evaporated and redissolved in 100 mL of water at pH 7.0 and a further extraction with ethyl acetate (three times with 100 mL of ethyl acetate each) was performed. The ethyl acetate phase is the FII fraction. The aqueous residue from this extraction was adjusted to pH 2.0 and extracted again with ethyl acetate (three times with 100 mL of ethyl acetate each) to obtain the FIII fraction (ethyl acetate phase) and FIV fraction (water phase). Ethyl acetate phases were evaporated under a nitrogen stream and redissolved in ethanol. All fractions were sealed under nitrogen and stored at −20 °C for further analysis.

### 4.5. Chemical Determinations

Total phenolic content of each sample was estimated using the modified method of Singleton and Rossi [33]. Briefly, samples were dried under a stream of nitrogen and then were dissolved in 3.5 mL water. Then, 0.1 mL of the Folin–Ciocalteu reagent was added to the test tubes, followed by 0.4 mL of 35% *w/v* sodium carbonate after 3 min. Gallic acid was used for the standard curve construction. The reaction mixture was rested for 1 h in the dark and the intensity of blue color was measured at 725 nm. The results were expressed as μg of gallic acid equivalents (GAE) per 100 μg of wine extract.

Ortho-diphenolic content was estimated according to the method described by Arranz et al. [34], with modifications. Briefly, samples were dried under a stream of nitrogen and dissolved in 0.1 mL ethanol. Then, 1.15 mL of a 1.087% *w/v* solution of sodium molybdate dehydrate was added. Standards of quercetin were prepared in the same way for the construction of the standard calibration curve. The reaction mixture was rested for 15 min at room temperature. The results were expressed as μg of quercetin equivalents per 100 μg of wine extract.

Sugar determination was performed according to the orcinol–sulphuric acid reaction [35]. Briefly, samples were dried under a stream of nitrogen and dissolved in 1 mL of water, 2 mL of orcinol (2 mg/mL in 70% sulfuric acid, *v/v*) was added and the solution was vigorously stirred. The reaction mixture was heated for 20 min at 80 °C and then was cooled down to room temperature. The absorbance was measured at 505 nm. Standards of glucose, from 10 to 100 µg, were prepared in the same way for the construction of the standard calibration curve.

Phosphorus was determined using magnesium nitrate and malachite green solution [36]. A volume of 30 μL of Mg(NO_3_)_2_•6H_2_O 10% *w/v* solution was added in the samples, in a glass testing tube. The mixture was heated to 85–100 °C in a water bath until the solvents were fully evaporated. Then, the tubes were heated at the top of the Bunsen burner flame for 15 sec and for a further 10 sec inside the flame. In the samples 1 mL of HCl was added and the mixture solution were heated for 15 min at 90–95 °C in a water bath and then cooled. Afterwards, 2 mL of malachite green solution (0.3% *w/v*) with (NH_4_)_6_MoO_27_•4H_2_O 4.2% *w/v* was added in the tube followed by 30 µL of Triton X-100 1 % w/v solution for color stabilization. The tubes were left for 35 min at room temperature and the absorbance was measured at 650 nm. A solution of potassium phosphate monobasic was used for the construction of the standard curve. The results were expressed as mg of phosphorus equivalents per 100 g of extract.

### 4.6. Soybean Lipoxygenase Inhibition Assay

The assay was performed according to a previously described procedure [37], with some modifications. The incubation mixture consisted of the appropriate amount (up to 40 μL) of the sample solution in the chosen solvent (water or dimethyl sulfoxide), 5 μL of the enzyme solution (60 units/μL in boric acid buffer) and 165 μL of 0.2 M boric acid buffer, pH 9.0. After incubation at room temperature for 5 min in the dark, the reaction was started by adding 40 μL of linoleic acid solution (937 μM in 25 μL dimethyl sulfoxide and 1225 μL buffer). The total volume of the reaction solution was 250 μL and the final concentration of linoleic acid was 150 μM in the reaction mixture. The conversion of linoleic acid to 13-hydroperoxylinoleic acid was recorded at 234 nm (room temperature) and compared to the appropriate standard solution, which did not contain the extracts. Every sample was tested in duplicate at several concentrations. The results were expressed as the IC_50_ values (mg/mL of the reaction mixture), giving the extract concentration needed to achieve 50% inhibition of the lipoxygenase activity.

### 4.7. Linoleic Acid Peroxidation Assay

This assay was performed according to the method of Choi et al. with modifications [38]. Briefly, the reaction mixture contained 500 μL linoleic acid (20 mM), 30 μL Tris HCl (100 mM, pH 7.5), 10 μL of ascorbic acid (20 mM) and an amount of 500 or 1000 mg of each wine extract in adequate solvent (ethanol or water). Linoleic acid peroxidation was initiated by the addition of 10 μL FeSO_4_• 7H2O (40 mM), was incubated for 10 min at 37 °C and was terminated by the addition of 103.5 μL ΤCA (40% *v/v*). An amount of 280 μL of TBA (1% *w/v* in 50 mM NaOH) was added to 0.7 mL of the reaction mixture, followed by heating at 95 °C for 10 min. The mixtures were centrifuged at 3500× *g* for 10 min. The absorbance of thiobarbituric acid-reacting substances (TBARS) was measured at 532 nm and converted into the percentage of antioxidant activity. The results are expressed as % inhibition when 500 or 1000 μg of each fraction was used.

### 4.8. Determination of DPPH Radical-Scavenging Activity

The DPPH assay was used to measure the free radical-scavenging capacity of the wine extracts, according to a previously reported method [39], with modifications. The appropriate amount of each sample, diluted in ethanol, was mixed with 35 μL of a freshly prepared ethanolic solution of 0.4 mg/mL DPPH in microplate wells. The total volume of the assay was 0.2 mL. The solutions were incubated at 37 °C for 30 and 60 min and the absorbance were measured at 492 nm with a microplate reader. The percentage of inhibition was calculated.

### 4.9. Inhibition of Cu^2+^ Induced Serum Oxidation

Fasting venous blood was collected in tubes without an anticoagulant and was left for 40 min at room temperature to clot. The blood was centrifuged at 1500× *g* for 10 min. The supernatant was collected and diluted with phosphate buffer solution at 1/12 ratio. The reaction mixture contained wine fraction samples in ethanol or water, 20 μL of human serum and 230 μL of copper sulfate 20 μM. The conjugated dienes’ absorption was measured continuously for 6 h at 245 nm at 37 °C. The analysis was conducted with the use of a microplate spectrophotometer (BioTek PowerWave XS2. Results are expressed in minutes (lag time).

### 4.10. Peripheral Blood Mononuclear Cells (PBMCs) Stimulation Assay

For the isolation of PBMCs blood was draught from a healthy in a vacutainer tube with EDTA. Blood was diluted 1:1 with a phosphate buffer solution (PBS) pH 7.4, carefully layered on LymphoprepTM and centrifuged to obtain the intermediate layer containing the PBMCs. Cells were washed with PBS pH 7.4, lysis buffer (155 mM NH_4_Cl, 0.15 M KHCO_3_ and 0.1 mM EDTA) and RPMI 1640 to lyse unwanted red blood cells. PBMC were resuspended in cell culture medium containing RPMI 1640, 10% *v/v* newborn calf serum, L-glutamine (2 mM) and 0.01% *w/v* of penicillin and streptomycin.

PBMCs (2 × 10^6^ cells/mL) were preincubated in 12 well plates for 1 h in 37 °C in the presence of various concentrations of TL, FI, FII, FIII or FIV extracts and then LPS was added in a final concentration of 100 ng/mL. After a 24 h incubation, cells were centrifuged, the supernatant was collected and stored at −80 °C until cytokines measurement and the sediment/pellet was kept at −20 °C for the calculation of total protein with the Bradford method [40]. An MTT (3-(4,5-Dimethylthiazol-2-yl)-2,5-diphenyltetrazolium bromide) viability test was run for all extracts prior to stimulated PBMC experiments so as to detect the maximum allowable concentration. The concentrations of secreted Il-1β and TNF-α were measured in the supernatant with the ELISA method (DuoSet ELISA kit, R&D Systems). The analysis was conducted using a microplate spectrophotometer (BioTek PowerWave XS2). Results were normalized with total protein content and expressed as % inhibition of LPS action.

### 4.11. Evaluation of the Extracts’ Anti-Platelet Properties by Light Transmission Aggregometry

Blood from apparently healthy volunteers was collected in a vacutainer with citrate and was centrifuged at 140× *g* without braking for 16 min and the supernatant platelet-rich plasma (PRP) was collected. Platelet-poor plasma (PPP) was prepared by recentrifugation of the pellet at 1500× *g* with braking for 15 min. Platelet count of PRP was adjusted to 500.000 per μL with the addition of PPP.

PRP (0.250 mL) was preincubated for 5 min in the presence or the absence (only the corresponding solvent) of extracts, at 37 °C with a stirring rate of 1000 rpm. After 5 min the agonist, namely PAF, ADP, TRAP, collagen or arachidonic acid (AA), was added. The extracts were dissolved in DMSO (1.6% maximum DMSO final concentration) or water. Aggregation responses were determined by the light transmittance method (Chronolog Aggregometer). Amplitude-optical aggregation results were expressed as a percentage of aggregation at a given time interval from agonist addition; aggregation was defined as the difference between the 0% (PRP) baseline and the 100% (PPP) baseline. The % inhibition of platelet aggregation induced by the extracts was calculated considering 0% inhibition of the platelet aggregation without the addition of the examined extract.

### 4.12. Calculation of the Biological Score

In order to estimate the pleiotropic biological activity of the extracts against oxidative stress, thrombosis and inflammation, a biological score was calculated. Initially, the anti-oxidant, anti-thrombotic and anti-inflammatory biological scores were calculated separately, adding up to the total biological score. More specifically, in the case that the biological activity was expressed as % inhibition score 0 was assigned when the value was <0 (activation), score 1 was assigned when the value was in the range of 0–25%, score 2 was assigned when the value was in the range of 25–50%, score 3 was assigned when the value was in the range of 50–75 μg and score 4 was assigned when the value was in the range of 75–100%. In the case of lag-time, where a higher value represents a more potent extract, score 1 was assigned when the value was in the range of 0-25 min/μg, score 2 was assigned when the value was in the range of 25–50 min/μg, score 3 was assigned when the value was in the range of 50–250 min/μg and score 4 was assigned when the value was >250 min/μg. In the case that biological activity is expressed as IC_50_ values, where lower values represent a more potent extract, the scores were assigned on a reverse scale, e.g., score 4 was assigned when the value was in the range of 0–25 μg, score 3 was assigned when the value was in the range of 25–50 μg, score 2 was assigned when the value was in the range of 50–250 μg and score 1 was assigned when the value was >250 μg. Based on the above, a score value was assigned for each individual experimental value and the results are expressed as the mean value of each score. Therefore each score ranges from 0 to 4.

### 4.13. HPLC Separation and Quantification of Phenolic Compounds

Millex^®^-SV syringeless PVDF filter membranes (5.0 μm; Millipore, Bedford, MA, USA) were used for the filtration of wine samples prior to instrumental analysis. The qualitative and quantitative analyses for the determination of individual phenolic compounds were carried out using an Agilent 1260 HPLC system interfaced with an Agilent 1260 diode array detector (DAD). Chromatographic separation was performed by Zorbax Eclipse Plus C18 (4.6 × 250 mm, 5 μm) column from Agilent. Data were acquired with the OpenLAB CDS Chemstation software package (Agilent Technologies). A previously chromatographic method was applied with some modifications [41]. More specifically, a gradient elution program with water:acetic acid:methanol (88:2:10, *v/v/v*; solvent A) and water:acetic acid:methanol (8:2:90, *v/v/v*; solvent B) as binary mobile phase mixture at a flow rate of 1 mL/min was used. The gradient elution started with 0% (*v/v*) Solvent B and increased linearly to 15% in 15.0 min, then to 50% in 10.0 min and to 70% in 10.0 min, held at 70% for 10.0 min, reverted to 100% in 10 min and re-equilibrated for 10.0 min (from 55.0 to 65.0 min) at 0% Solvent B for a total run time of 65.0 min. Sample and standards were injected on column with full-loop injection (20 μL) and the column temperature was set at 30 °C. The detection wavelength was 280 nm for gallic acid, catechin and tyrosol, while for resveratrol and chlorogenic acid was 320 nm and 365 nm for quercetin and standards curves were performed. The concentration of phenolic compounds in the FII extracts of one white (Robola of Kefalonia) and one red (Vertzami) wine, from three independently experiments, is expressed as μM and presented in the text.

### 4.14. Statistical Analysis

Normality was tested with the Kolmogorov–Smirnoff criterion. All the variables had normal distribution and the results were expressed as mean values ± SEM. For the comparison of the fractions or the type of wines (red versus white) a one-way analysis of variance (ANOVA) was performed and a post hoc analysis was carried out, where appropriate, with the Bonferroni correction. Pearson partial correlation coefficients were evaluated. The level of significance used was 5%. SPSS v21 software was used for statistical analysis. PCA analysis was performed with SIMCA v.14.1 (Umetrics) using both unsupervised (PCA) and supervised (PLS-DA) methods. The variables were transformed to Log10. The generated models were validated through a permutation test with 100 iterations.

## Figures and Tables

**Table 1:** Chemical determinations in wines per extract type and per wine color.

	Extract	Total Phenolic Compounds (μg GA/100 μg Extract)	Ortho-Phenolic Compounds (μg Quercetin/100 μg Extract)	μg PHOSPORUS/100 μg Extract	μg Glucose/100 μg Extract
White wines	TL	4.32 ± 0.92 ^a^	2.61 ± 0.87 ^a^	0.014 ± 0.001^a^	5.84 ± 2.11 ^a^
	FI	1.05 ± 0.13 ^a^	0.47 ± 0.08 ^a^	0.277 ± 0.035 ^b^	19.33 ± 2.72 ^b^
	FII	24.40 ± 2.62 ^b^	9.62 ± 2.27 ^b^	0.019 ± 0.002 ^a^	6.89 ± 0.65 ^a^
	FIIΙ	11.44 ± 1.41 ^c^	14.07 ± 2.23 ^b^	0.010 ± 0.001 ^a^	0.99 ± 0.16 ^a^
	FIV	0.58 ± 0.04 ^a^	0.48 ± 0.07 ^a^	0.036 ± 0.004 ^a^	1.44 ± 0.24 ^a^
Red wines	TL	5.18 ± 0.40 ^a^	2.11 ± 0.23 ^a^	0.011 ± 0.002 ^a^	4.67 ± 0.95 ^a^
	FI	2.65 ± 0.57 ^a,^*	0.53 ± 0.14 ^a^	0.241 ± 0.035 ^b^	45.70 ± 12.45 ^b^
	FII	29.64 ± 4.30	13.68 ± 2.37 ^a^	0.031 ± 0.005 ^a,^*	15.07 ± 2.11 ^a,^*
	FIIΙ	12.84 ± 1.31 ^a^	8.61 ± 1.32 ^b,^*	0.012 ± 0.002 ^a^	3.24 ± 0.44 ^a,^*
	FIV	8.72 ± 5.18 ^a^	1.28 ± 0.58 ^a^	0.040 ± 0.006 ^a^	46.88 ± 30.50 ^a^

Within each type of wine different letters indicate significant differences between extracts (*p* < 0.05). * *p* < 0.05 versus white wines based on a one way ANOVA.

**Table 2:** Anti-oxidant activity of wines per extract type and per wine color.

	Extract	DPPH Scavenging Activity (IC_50_, μg)	LOX-Inhibition (IC_50_, μg)	Fe-induced Linoleic Acid Oxidation Inhibition (% Inhibition)	Cu-induced Serum Oxidation Inhibition (min/μg)
White wines	TL	118.4 ± 24.4 ^a^	131.5 ± 49.0 ^a^	44.9 ± 25.0 ^a^	84.0 ± 20.2 ^a,b^
	FI	1068.9 ± 215.2 ^b^	1963.3 ± 271.4 ^b^	36.8 ± 17.8 ^a^	8.3 ± 1.6 ^a,b^
	FII	27.5 ± 4.2 ^a^	34.4 ± 6.0 ^a^	33.9 ± 27.7 ^a^	214.4 ± 53.2 ^c^
	FIIΙ	28.1 ± 6.1 ^a^	137.0 ± 23.9 ^a^	62.3 ± 8.6 ^a^	160.5 ± 17.8 ^a,c^
	FIV	385.3 ± 49.1 ^a^	nd	27.5 ± 11.4 ^a^	7.6 ± 0.8 ^a,b^
Red wines	TL	86.8 ± 12.8 ^a^	161.0 ± 28.8 ^a^	38.7 ± 14.9 ^a^	69.9 ± 11.1 ^a^
	FI	85.4 ± 28.5 ^a,^*	453.2 ± 90.8 ^b,^*	42.8 ± 12.5 ^a^	20.4 ± 3.3 ^a,^*
	FII	71.3 ± 16.9 ^a,^*	66.8 ± 8.0 ^a,^*	67.1 ± 9.6 ^a^	647.9 ± 98.0 ^b,^*
	FIIΙ	18.5 ± 4.9 ^a^	188.4 ± 33.6 ^a^	36.5 ± 25.4 ^a^	129.9 ± 9.4 ^a^
	FIV	710.6 ± 314.3 ^b^	nd	11.6 ± 15.5 ^a^	89.7 ± 39.5 ^a,^*

Within each type of wine different letters indicate significant differences between extracts (*p* < 0.05). * *p* < 0.05 versus white wines based on a one way ANOVA. nd: not detected.

**Table 3:** Anti-platelet activity of wines per type of extract and per color of wine.

	Extract	PAF (% Inhibition)	ADP (% Inhibition)	TRAP (% Inhibition)	Collagen (% Inhibition)	AA (% Inhibition)
White wines	TL	58.1 ± 11.8 ^a^	64.6 ± 8.5 ^a^	−3.7 ± 15.5 ^a^	70.1 ± 10.6 ^a^	60.7 ± 10.3 ^a^
	FI	13.5 ± 2.2 ^b^	18.7 ± 5.0 ^b^	22.2 ± 12.2 ^a^	7.9 ± 4.1 ^b^	−3.8 ± 3.8 ^b^
	FII	53.5 ± 9.2 ^a^	67.0 ± 12.7 ^a^	27.9 ± 8.6 ^a^	63.6 ± 18.7 ^a^	91.8 ± 5.5 ^a^
	FIIΙ	49.8 ± 10.6 ^a^	64.6 ± 14.3 ^a^	19.1 ± 6.0 ^a^	69.8 ± 11.4 ^a^	54.6 ± 7.9 ^a^
	FIV	12.6 ± 14.3 ^b^	23.7 ± 4.0 ^b^	−24.5 ± 19.0 ^a^	14.5 ± 5.7 ^b^	10.4 ± 9.2 ^b^
Red wines	TL	61.6 ± 12.0 ^a^	62.2 ± 11.2 ^a^	26.2 ± 7.6 ^a^	68.0 ± 11.6 ^a^	64.2 ± 10.0 ^a^
	FI	57.6 ± 16.5 ^a,^*	46.6 ± 10.4 ^a,^*	51.7 ± 10.0 ^a,^*	42.5 ± 14.3 ^a,^*	36.3 ± 15.5 ^a,b,^*
	FII	60.1 ± 13.2 ^a^	62.8 ± 11.7 ^a^	59.6 ± 10.3 ^a,^*	66.4 ± 15.1 ^a^	87.2 ± 8.6 ^a,b^
	FIIΙ	36.5 ± 12.3 ^a^	63.1 ± 7.0 ^a^	25.4 ± 6.3 ^a^	61.7 ± 11.0 ^a^	56.8 ± 19.5 ^a^
	FIV	43.9 ± 14.1 ^a^	33.0 ± 8.3 ^a^	26.0 ± 13.5 ^a,^*	29.7 ± 12.0 ^b^	2.3 ± 5.8 ^c^

Within each type of wine different letters indicate significant differences between extracts (*p* < 0.05). * *p* < 0.05 versus white wines based on a one way ANOVA.

**Table 4:** Anti-inflammatory activity of wines per extract type and per wine color.

Type of Wine		TNF-α % Inhibition		Il-1β % Inhibition	
		500 μg	100 μg	500 μg	100 μg
White wines	TL	0.8 ± 44.9 ^a^		5.1 ± 34.8	
	FI		18.6 ± 8.2 ^a^		7.8 ± 9.5 ^a^
	FII	76.4 ± 34.7 ^b^		63.1 ± 29.7	
	FIIΙ	32.2 ± 17.3 ^a^		23.1 ± 21.5	
	FIV		27.1 ± 10.7 ^b^		11.9 ± 11.0 ^a^
Red wines	TL	18.0 ± 14.3 ^a^		22.7 ± 18.3 ^a^	
	FI		24.1 ± 22.8 ^a^		14.3 ± 20.5 ^a^
	FII	51.6 ± 49.9 ^b^		43.6 ± 28.7 ^a,^*	
	FIIΙ	24.9 ± 24.9 ^a^		33.6 ± 17.4 ^a^	
	FIV		21.4 ± 14.9 ^a^		13.9 ± 13.2 ^a^

Within each type of wine different letters indicate significant differences between extracts (*p* < 0.05). * *p* < 0.05 versus white wines based on a one way ANOVA.

**Table 5:** Total biological score and per mechanism.

	Extract	Anti-Oxidant	Anti-Platelet	Anti-Inflammatory	Total
White	TL	2.3 ± 0.2 ^a^	2.7 ± 0.2 ^a^	1.2 ± 0.2 ^a^	2.0 ± 0.1 ^a^
	FI	1.2 ± 0.2 ^b^	1.3 ± 0.1 ^b^	1.1 ± 0.1 ^a^	1.2 ± 0.1 ^b^
	FII	3.2 ± 0.2 ^c^	2.5 ± 0.3 ^a^	3.3 ± 0.2 ^b^	3.1 ± 0.1 ^c^
	FIIΙ	2.9 ± 0.1 ^c^	2.6 ± 0.3 ^a^	1.5 ± 0.1 ^a^	2.2 ± 0.1 ^a^
	FIV	1.1 ± 0.1 ^b^	1.2 ± 0.1 ^b^	1.3 ± 0.1 ^a^	1.3 ± 0.0 ^b^
Red	TL	2.3 ± 0.1 ^a,b^	2.6 ± 0.3 ^a,b^	1.4 ± 0.1 ^a^	1.9 ± 0.1 ^a^
	FI	1.8 ± 0.2 ^a,b,^*	2.3 ± 0.3 ^a,b,^*	1.3 ± 0.2 ^a^	1.6 ± 0.2 ^a,^*
	FII	3.2 ± 0.1 ^c^	2.8 ± 0.3 ^a^	2.2 ± 0.2 ^b,^*	2.6 ± 0.2 ^b,^*
	FIIΙ	2.8 ± 0.1 ^a,c^	2.5 ± 0.2 ^a,b^	1.6 ± 0.2 ^a,b^	2.2 ± 0.2 ^a,b^
	FIV	1.7 ± 0.1 ^a,b^	1.7 ± 0.2 ^b^	1.3 ± 0.1 ^a^	1.6 ± 0.1 ^a,^*

Within each type of wine different letters indicate significant differences between extracts (*p* < 0.05). * *p* < 0.05 versus white wines based on a one way ANOVA.

**Table 6:** Correlations of chemicals determinations and biological scores per type of extract.

Extract	Action	Total Phenolic Compounds	Ortho-Phenolic Compounds	Phosphorus	Sugars
TL	Anti-oxidant	0.585 **	0.448 *	ns	ns
	Anti-platelet	ns	ns	0.487 *	−0.427 *
	Anti-inflammatory	ns	−0.790 *	−0.869 **	ns
	Total	ns	ns	ns	ns
FI	Anti-oxidant	ns	0.457 *	ns	0.741 ***
	Anti-platelet	0.739 ***	0.486 *	ns	0.678 **
	Anti-inflammatory	ns	−0.477 *	−0.432 *	ns
	Total	0.555 **	ns	ns	0.377 *
FII	Anti-oxidant	ns	ns	ns	ns
	Anti-platelet	0.548 *	ns	0.505 *	ns
	Anti-inflammatory	ns	0.614 *	ns	ns
	Total	0.472 *	0.389 *	ns	0.420 *
FIV	Anti-oxidant	0.911 **	0.852 **	0.561 **	0.908 **
	Anti-platelet	ns	ns	0.447 *	ns
	Anti-inflammatory	ns	ns	ns	ns
	Total	0.779 ***	0.780 ***	0.574 **	0.787 ***

* Correlation is significant at the 0.05 level (2-tailed); ** correlation is significant at the 0.005 level (2-tailed); *** correlation is significant at the <0.000 level (2-tailed); ns: not significant.

## Supplementary Materials

The following are available online, Table S1: Chemical determinations in wine extracts per variety, Table S2: Anti-oxidants assays in wine extracts per variety, Table S3: Anti-platelet activity of the extracts per variety, Table S4: Anti-inflammatory activity of the extracts per variety.
