# Supplementary material for: Evaluation of Anti-Inflammatory, Anti-Platelet and Anti-Oxidant Activity of Wine Extracts Prepared from Ten Different Grape Varieties
Source: Molecules. 2020 Oct 30;25(21):5054. doi: 10.3390/molecules25215054 (PMC7662244; doi:10.3390/molecules25215054)
Supplement: Supplementary file 1 [file molecules-25-05054-s001.pdf]

Supplementary Tables

Table S1. Chemical determinations in wine extracts per variety

Variety	Extract	Total phenolic compounds (μg GA/100μg extract)	Ortho-phenolic compounds (μg quercetin/100 μg extract)	μg Phosporus /100 μg extract	μg Glucose /100 μg extract
Robola of Kefalonia	TL	1.98±0.23 [a]	0.43±0.02 [a]	0.010±0.002[a]	2.72±0.08[a]
	FI	1.05±0.33 [a]	0.31±0.01 [a]	0.277±0.030[b]	23.63±0.32[b]
	FII	18.24±2.31 [b]	4.43±0.05 [b]	0.017±0.001[a]	5.56±0.3[c]
	FIII	6.09±0.99 [c]	7.26±0.50 [c]	0.007±0.001[a]	0.50±0.16[d]
	FIV	0.43±0.02 [a]	0.22±0.01 [a]	0.042±0.001[c]	2.86±0.07[a]
Tsaousi	TL	3.16±0.32 [a]	0.73±0.02 [a]	0.014±0.002 [a]	1.65±0.01[a]
	FI	0.86±0.03 [a]	0.31±0.00 [a]	0.368±0.056 [b]	13.63±1.75[b]
	FII	30.45±3.90 [b]	3.54±0.36 [b]	0.031±0.003 [a]	5.06±0.78[c]
	FIII	10.38±0.94 [c]	13.00±0.69 [c]	0.004±0.001 [a]	0.49±0.04[a]
	FIV	0.51±0.00 [a]	0.39±0.01 [a]	0.024±0.001 [a]	0.87±0.06[a]
Kakotrigis	TL	7.11±0.97 [a]	7.03±0.23 [a]	0.022±0.001 [a]	3.10±0.13[a]
	FI	1.88±0.13 [a]	0.79±0.01 [a]	0.438±0.027 [b]	14.02±0.13[b]
	FII	36.63±0.23 [b]	20.50±2.31 [b]	0.029±0.000 [a]	8.71±0.18[c]
	FIII	17.21±3.75 [c]	24.91±2.23 [b]	0.019±0.002 [a]	1.33±0.05[d]
	FIV	0.65±0.01 [a]	0.45±0.00 [a]	0.037±0.000 [a]	1.04±0.13[d]
Muscat of Kefalonia	TL	1.30±0.00 [a]	0.56±0.02 [a]	0.006±0.001 [a]	18.40±1.41 [a]
	FI	0.44±0.01 [a]	0.05±0.01 [a]	0.100±0.001 [b]	37.75±0.21 [b]
	FII	19.04±0.06 [b]	4.97±0.08 [b]	0.014±0.001 [a]	9.70±0.28 [c]
	FIII	7.90±0.95 [c]	20.58±2.80 [c]	0.011±0.001 [a]	1.90±0.00 [d]
	FIV	0.76±0.03 [a]	0.87±0.09 [a, b]	0.045±0.013 [c]	2.15±0.21 [d]
White thiako	TL	8.05±0.49 [a]	5.35±0.32 [a]	0.012±0.002 [a]	3.35±0.07 [a]
	FI	1.03±0.12 [b]	0.69±0.05 [b]	0.175±0.043 [b]	13.48±0.84 [b]
	FII	17.65±0.07 [c]	14.65±0.01 [c]	0.010±0.001 [a]	5.45±0.07 [a]
	FIII	17.17±0.71 [c]	8.00±0.17 [d]	0.010±0.002 [a]	1.25±0.07 [a, d]
	FIV	0.57±0.01 [b]	0.47±0.07 [b]	0.030±0.028 [a]	0.66±0.04 [d]
Petrokoritho	TL	5.05±0.07 [a]	1.78±0.08 [a]	0.023±0.003 [a]	2.80±0.00 [a]
	FI	1.03±0.12 [b]	0.41±0.04 [b]	0.155±0.041 [b]	12.28±0.78 [b]
	FII	11.00±0.57 [c]	1.67±0.10 [a]	0.030±0.000 [a]	4.25±0.07 [a, d]
	FIII	7.05±0.21 [d]	4.83±0.36 [c]	0.007±0.001 [c]	1.30±0.14 [d]
	FIV	0.55±0.01 [b]	0.45±0.01 [b]	0.030±0.005 [a]	0.53±0.07
Vertzami	TL	6.99±0.56 [a]	2.83±0.09 [a]	0.012±0.002 [a]	3.56±0.16 [a]
	FI	4.24±0.17 [a, b]	1.45±0.12 [a, b]	0.466±0.040 [b]	149.57±1.25 [b]
	FII	54.49±1.99 [c]	13.38±0.13 [c]	0.053±0.009 [a]	25.61±1.02 [c]
	FIII	18.12±1.21 [d]	16.14±0.75 [d]	0.011±0.001 [a]	2.53±0.25 [a]
	FIV	1.57±0.11 [b]	0.53±0.03 [b]	0.017±0.001 [a]	1.26±0.04 [a]
Avgoustiatis	TL	4.25±0.53 [a]	0.76±0.03 [a]	0.006±0.000 [a]	10.06±0.08 [a]

	FI	2.04±0.83 [a]	0.38±0.03 [a]	0.174±0.019 [b]	48.04±2.37 [b]
	FII	27.76±2.45 [b]	5.04±0.33 [b]	0.029±0.003 [a]	14.45±1.58 [a]
	FIII	8.90±0.94 [c]	6.93±0.48 [c]	0.015±0.000 [a]	4.36±0.17 [a]
	FIV	0.81±0.49 [a]	0.08±0.00 [a]	0.024±0.001 [a]	4.28±0.15 [a]
Red thiako	TL	3.30±0.14 [a]	2.15±0.21 [a]	0.007±0.002 [a]	2.00±0.14 [a]
	FI	0.11±0.00 [b]	0.12±0.01 [a]	0.151±0.073 [b]	0.11±0.00 [b]
	FII	32.40±1.56 [c]	20.43±2.03 [b]	0.010±0.001 [a]	17.27±1.01 [c]
	FIII	10.80±0.85 [d]	11.14±0.24 [c]	0.017±0.014 [a]	3.05±0.07 [a]
	FIV	1.50±0.10 [a, b]	0.57±0.06 [a]	0.053±0.006 [b]	1.65±0.07 [a]
Mavrodaphne of Kefalonia	TL	5.93±0.38 [a]	2.83±0.20	0.008±0.002 [a]	4.95±0.07 [a]
	FI	5.73±0.29 [a]	0.37±0.03	0.370±0.014 [b]	18.50±0.29 [b]
	FII	24.90±0.46 [b]	20.91±0.83	0.034±0.006 [b, c]	12.70±0.57 [c]
	FIII	14.05±0.07 [c]	5.92±0.50	0.013±0.001 [a, c]	4.95±0.07 [a]
	FIV	50.70±3.25 [d]	4.77±0.20	0.069±0.006 [d]	273.05±0.78 [d]

Within each wine different letters indicate significant differences between extracts based on one way ANOVA.

Table S2. Anti-oxidants assays in wine extracts per variety

Variety	Extract	DPPH scavenging activity	LOX-inhibition	Fe-induced Linoleic acid oxidation inhibition	Cu-induced Serum oxidation inhibition
Robola of Kefalonia	TL	215.3±16.7[a]	82.5±7.9 [a]	50.7±8.2[a]	34.1±6.2 [a]
	FI	331.8±88.0 [a]	nd	55.6±9.2 [a]	4.2±0.1 [a]
	FII	33.1±1.6 [b]	23.7±2.4 [b]	92.3±11.3 [b]	64.7±39.0 [a]
	FIII	27.8±9.9 [b]	91.6±6.2 [a]	44.5±5.2 [a]	174.5±25.8 [b]
	FIV	473.0±191.1 [a]	nd	43.8±5.8 [a]	4.6±0.4 [a]
Tsaousi	TL	163.5±4.0 [a]	nd	28.1±3.8 [a]	39.1±0.9 [a]
	FI	2070.4±53.3 [b]	nd	82.0±11.9 [b]	4.6±0.1 [b]
	FII	26.7±0.0 [c]	17.8±5.7	42.9±7.2 [a]	98.2± [c]
	FIII	17.1±0.1 [c]	nd	92.8±12.5 [b]	117.8±12.0 [c,]
	FIV	263.3±37.5 [a]	nd	24.1±2.8 [a]	6.5±1.5 [b]
Kakotrigis	TL	45.7±19.9 [a]	nd	26.8±4.1 [a]	164.1±1.2 [a, b]
	FI	1296.4±11.5 [b]	890.1±121.3 [a]	33.0±4.8 [a]	14.5±0.4 [a, b]
	FII	11.2±5.9 [a]	40.1±1.4 [b]	93.3±13.8 [b]	377.7±151.6 [b]
	FIII	60.4±2.5 [a]	128.5±7.6 [b]	58.0±6.5 [b]	239.4±21.3 [a, b]
	FIV	500.1±8.1 [b]	nd	73.9±13.8 [b]	7.0±1.0 [a, b]
Muscat of Kefalonia	TL	103.2±4.9 [a]	nd	82.2±10.9 [a]	31.1±0.6 [a]
	FI	1242.7±4.7 [b]	nd	14.2±3.9 [b]	4.6±2.1 [a, b]
	FII	19.4±3.0 [c]	88.6±12.3	79.5±10.5 [a]	114.9±38.5 [c]
	FIII	28.1±10.5 [c]	nd	77.5±9.9 [a]	7.0±1.3 [b]
	FIV	472.4±1.5 [d]	nd	17.4±2.3 [b]	31.1±0.6 [a]
White thiako	TL	15.8±1.2 [a]	180.5±±24.3 [a]	55.2±7.8 [a]	151.7±9.1 [a]
	FI	403.3±120.2 [b]	nd	80.3±11.9 [a]	13.6±2.1[b]
	FII	47.4±0.9 [a]	61.4±9.4 [a]	29.8±4.2 [b]	235.3±57.3 [a]

	FIII	7.3±0.0 ^{a}	nd	26.7±5.6 ^{b}	116.9±31.7 ^{a}
	FIV	174.0±82.8 $^{a, b}$	nd	73.2±8.9 ^{a}	11.4±2.1 ^{b}
Petrokoritho	TL	121.3±8.3 $^{a, b}$	nd	80.5±13.2 ^{a}	33.5±4.3 ^{a}
	FI	144.0±1.6 $^{a, b}$	nd	59.5±5.6 ^{b}	8.8±1.0 ^{a}
	FII	29.2±4.9 ^{b}	93.7±12.2 ^{a}	80.0±11.8 ^{a}	703.1±206.9 ^{b}
	FIII	50.5±8.1 ^{b}	202.7±72.5 ^{a}	92.9±14.3 ^{a}	144.7±3.7 ^{a}
	FIV	312.0±101.3 ^{a}	nd	38.2±4.5 ^{b}	17.1±5.8 ^{a}
Vertzami	TL	24.1±12.4 ^{a}	nd	64.9±8.1 ^{a}	139.7±6.3 ^{a}
	FI	13.7±6.9 ^{a}	199.0±26.2 ^{a}	77.0±10.9 ^{a}	34.3±12.2 ^{b}
	FII	93.5±0.6 ^{b}	42.5±5.9 ^{b}	69.4±8.9 ^{a}	417.9±115.0 ^{b}
	FIII	9.8±4.4 ^{a}	135.7±7.3 ^{a}	56.7±7.8 ^{a}	155.1±13.6 ^{a}
	FIV	139.8±14.2 ^{c}	nd	36.3±4.4 ^{a}	11.6±1.4 ^{b}
Avgoustiatis	TL	52.3±53.7 ^{a}	77.0±6.7 ^{a}	19.3±2.4 ^{a}	52.8±7.9 ^{a}
	FI	107.9±27.4 ^{a}	752.2±114.6 ^{b}	40.6±6.5 ^{b}	15.1±0.4 ^{a}
	FII	16.6±1.2 ^{a}	85.0±9.8 ^{a}	81.4±11.9 ^{b}	396.5±84.8 ^{b}
	FIII	160.4± ^{a}	63.3±5.8 ^{a}	55.0±7.8 ^{b}	105.4±9.6 ^{a}
	FIV	11.0±4.0 ^{b}	nd	10.7±2.5 ^{a}	14.9±5.0 ^{a}
Red thiako	TL	110.6±28.5 $^{a, b}$	nd	35.1±4.5 ^{a}	50.8±14.3 ^{a}
	FI	234.0±106.1 ^{a}	nd	69.2±8.5 ^{b}	16.8±2.2 ^{a}
	FII	47.8±5.8 $^{a, b}$	41.5±1.5 ^{a}	68.3±9.8 ^{b}	546.5±184.4 ^{b}
	FIII	12.1±4.9 ^{b}	145.3±33.5 ^{b}	68.2±7.4 ^{b}	144.0±42.7 ^{a}
	FIV	97.5±1.0 $^{a, b}$	nd	5.6±2.6 ^{c}	16.6±2.6 ^{a}
Mavrodaphne of Kefalonia	TL	70.0±6.6 ^{a}	239.4±57.6 ^{a}	90.6±11.2 ^{a}	108.9±6.7 ^{a}
	FI	54.9±0.4 ^{a}	408.2±83.8 ^{b}	43.6±5.8 ^{b}	32.7±330.1 ^{a}
	FII	25.4±6.6 ^{b}	67.2±14.1 ^{a}	76.7±12.6 ^{a}	1160.2±150.6 ^{b}
	FIII	12.4±2.2 ^{b}	337.2±73.3 ^{b}	75.9±14.3 ^{a}	105.4±34.7 ^{a}

FIV	18.4±8.5 ^{b}	nd	8.9±0.8 ^{c}	337.5±40.9 ^{a}

Within each wine different letters indicate significant differences between extracts based on one way ANOVA. nd: not detected

Table S3. Anti-platelet activity of the extracts per variety

Variety	Extract	PAF	ADP	TRAP	Collagen	AA
Robola of Kefalonia	TL	45.2±26.1[a]	79.8±8.1 [a]	0.0±1.9 [a]	93.2±6.8 [a]	76.4±5.0 [a]
	FI	13.5±3.6[b]	9.1±5.1 [b]	0.0± 1.5[a]	5.4±8.0 [b]	0.0±0.6 [b]
	FII	70.8±17.5 [a]	97.3±0.0 [a]	24.9±4.5 [b]	100.0±0.0 [a]	100.0±0.0 [a]
	FIII	57.3±27.3 [a]	94.7±1.1 [a]	0.0±1.4 [a]	100.0± 0.0 [a]	63.8±13.2 [a]
	FIV	9.3±2.4 [b]	13.9±3.3 [b]	22.2±3.9 [b]	16.5±7.9 [b]	3.1±3.1 [b]
Tsaousi	TL	76.8±6.8 [a]	91.6±0.2 [a]	1.8±6.9 [a]	83.4±3.4 [a]	19.5±10.9 [a]
	FI	7.2±1.5 [b]	7.9±2.8 [b]	0.0±4.9 [a]	1.0±2.2 [b]	0.0±5.8 [a]
	FII	50.4±27.6 [a]	46.1±28.1 [a]	14.8±7.6 [a]	100.0±0.0 [a]	100.0±0.0 [b]
	FIII	84.5±7.9 [a]	75.0±8.3 [a]	20.5±2.7 [a]	100.0±0.0 [a]	81.3±3.1 [b]
	FIV	1.3±0.8 [b]	17.6±7.4 [b]	17.8±6.9 [a]	9.1±4.3 [b]	7.6±3.8 [a]
Kakotrigis	TL	86.8±5.8 [a]	85.0±1.3 [a]	5.9±3.4 [a]	100.0±0.0 [a]	100.0±0.0 [a]
	FI	17.7±2.4 [b]	12.6±1.9 [b]	10.0±5.6 [a]	8.6±4.8 [b]	5.7±12.9 [b]
	FII	74.7±8.3 [a]	90.8±3.7 [a]	73.8±11.1	100.0±0.0 [a]	100.0±0.0 [a]
	FIII	26.0±23.1	89.7±0.8 [a]	27.5±15.2 [a]	100.0±0.0 [a]	42.3±2.0 [c]
	FIV	17.3±3.8 [b]	17.1±0.4 [b]	0.0±2.3[a]	14.6±8.0 [b]	0.0±2.3 [b]
Muscat of Kefalonia	TL	42.6±52.6 [a]	39.3±21.5 [a]	0.0±5.3 [a]	27.9±27.9 [a]	27.4±15.2 [a]
	FI	0.0±10.7	37.5±11.1 [a]	57.3±14.4 [b]	22.6±5.9 [a]	7.1±3.5 [b]
	FII	12.0±3.8 [b]	33.6±25.9 [a]	13.5±13.5 [b]	18.0±6.3[a]	75.0±20.3 [c]
	FIII	22.6±12.2 [b]	34.4±54.4 [a]	23.8±23.8 [b]	41.4±16.9 [a]	22.2±15.3 [a]
	FIV	16.0±5.4 [b]	0.0±2.8 [b]	0.0±7.4 [a]	2.8±2.3 [a]	34.4±13.8 [a]
White thiako	TL	48.6±5.3 [a]	52.1±16.2 [a]	44.9±20.2 [a]	54.1±2.0[a]	55.8±7.7 [a]
	FI	0.0±3.8 [b]	34.6±16.8 [a]	49.2±18.5 [a]	0.0±5.8 [b]	21.2±12.3 [b]
	FII	28.1±8.1 [a]	0.0±4.5 [b]	0.0±5.6 [b]	0.0±3.9 [b]	59.6±12.3[a]
	FIII	56.0±16.9 [a]	33.0±51.6 [a]	27.8±14.7 [a]	21.8±9.3 [a]	40.4±8.8[a]
	FIV	7.9±3.2 [b]	35.4±4.4 [a]	0.0±3.4 [b]	48.7±18.3 [a]	8.1±4.9 [b]

Petrokoritho	TL	88.3±6.1 a	84.0±3.0 a	58.3±16.5 a	76.0±7.9 a	67.7±5.4 a
	FI	0.0±5.8 b	33.1±9.7 b	37.3±4.0 a	16.2±5.2 b	7.9±3.2 b
	FII	60.7±18.7 a	15.7±11.6 b	35.9±2.9 a	84.6±10.2 a	87.0±20.0 a
	FIII	0.0±8.2 b	37.8±12.2 b	20.0±15.2 a	48.3±12.8 a	56.7±20.0
	FIV	84.5±20.5 a	35.2±27.1 b	36.2±35.2 a	48.9±11.7 a	2.3±16.1 b
Vertzami	TL	83.8±1.9 a	93.1±3.9 a	2.0±2.0 a	99.4±0.6 a	94.4±5.6 a
	FI	100.0±0.0 a	95.9±4.1 a	90.0±10.0 b	88.8±5.2 a	87.7±0.5 a
	FII	93.2±5.5 a	96.6±3.4 a	96.3±3.7 b	100.0±0.0 a	100.0± 0.0 a
	FIII	49.9±30.4 a	94.7±3.7 a	20.4±8.0 a	100.0±0.0 a	54.0±34.5 a
	FIV	7.8±3.2 b	6.9±3.5 b	22.2±13.8 a	23.2±14.3 b	0.0±4.7 b
Avgoustiatis	TL	56.9±18.5 a	87.4±4.5 a	17.7±0.2 a	91.2±8.8 a	13.5±16.3 a
	FI	12.3±1.5 a	9.6±5.6 b	4.4±2.1 a	9.2±6.0 b	0.0±12.8 a
	FII	81.3±17.2 a	99.0±1.0 a	72.8±2.2 b	100.0±0.0 a	100.0±0.0 b
	FIII	50.8±41.1 a	76.0±2.6 a	25.6±0.9 a	100.0±0.0 a	88.0±1.6 b
	FIV	9.1±3.5 b	15.7±7.3 b	0.0±5.9 a	6.1±2.9 b	1.4±2.0 a
Red thiako	TL	51.5±21.5 a	32.1±0.4 a	15.4±0.5 a	52.3±18.9 a	78.9±11.0 a
	FI	43.8±18.3 a	16.3±16.3 a	36.8±13.8 a	0.0±0.5 b	9.2±9.2 b
	FII	33.3±15.3 a	58.9±18.1 a	28.7±9.1 a	25.4±10.2 a	51.1±20.3 a
	FIII	24.2±24.2 a	44.4±13.2 a	39.9±18.8 a	32.9±29.6 a	0.0±0.9 b
	FIV	31.6±31.6 a	53.9±19.4 a	55.8±14.6 a	0.0±2.8 b	0.0±2.5 b
Mavrodaphne of Kefalonia	TL	83.3±6.8 a	5.3±3.4 a	21.8±0.9 a	13.3±3.3 a	66.8±17.9 a
	FI	88.7±7.3 a	72.7±8.2	66.5±5.4	39.0±15.8 b	82.4±15.3 a
	FII	5.4±8.5	35.5±11.5	22.9±5.6 a	10.7±0.8 a	71.9±14.5 a
	FIII	21.0±16.5	54.2±15.8	0.0±2.6	38.6±16.5 b	0.0±3.5 b
	FIV	71.4±14.6 a	31.0±13.7	0.0±8.6	49.9±29.0 b	20.3±20.3 b

Within each wine different letters indicate significant differences between extracts based on one way ANOVA.

Table S4. Anti-inflammatory activity of the extracts per variety

Variety	Extract	TNF-α		Il-1β	
		500μg,	100μg,	500μg,	100μg,
Robola of Kefalonia	TL	21.5±16.7[a]		31.0±15.2 [a]	
	FI		26.8±4.5		7.8±2.2
	FII	91.6±8.3 [b]		85.2±6.7 [a]	
	FIII	15.4±8.5 [a]		3.7±2.9 [a]	
	FIV		37.0±4.0		2.3.3±0.4
Tsaousi	TL	33.2±12.1 [a]		16.7±18.5 [a]	
	FI		16.3±0.9		0.2±5.4
	FII	0.0±1.1 [a]		9.2±2.6 [a]	
	FIII	50.4±9.4 [a]		9.1±6.1 [a]	
	FIV	-	25.9±0.6		22.6±2.8
Kakotrigis	TL	A [a]		A [a]	
	FI		21.9±2.0		11.7±1.4
	FII	89.3±7.9[b]		65.2±20.9 [b]	
	FIII	A		20.4±20.2	
	FIV		15.2±8.3		3.4±3.0
Muscat of Kefalonia	TL	22.9±11.7 [a]		42,7±13.9 [a]	
	FI		19.9±7.4		15.8±8.8
	FII	37.0±11.8 [a]		46.9±13.6 [a]	
	FIII	36.2±7.1 [a]		47.2±3.3 [a]	
	FIV		23.3±6.8		0.4±10.0
White thiako	TL	0,99±3.5 [a]		0,0±7.6	
	FI		3.4±5.2		7.8±6.9
	FII	103.9±8.9 [b]		92.2±26.1 [b]	

	FIII	17.8±9.7 ^{a}		51.8±6.6	
	FIV		33.5±12.3		18.0±6.2
Petrokoritho	TL	24.5±21.1 ^{a}		43.1±14.6 ^{a}	36.9±2.4
	FI		68.0±25.3		539.±20.3
	FII	A		20.5±8.7 ^{a}	
	FIII	13,3±34.4 ^{a}		58.6±13.3 ^{a}	
	FIV		24.5±7.2		16.4±9.0
Vertzami	TL	2,0±9.2 ^{a}		2,1±? ^{a}	
	FI		11.1±2.0		15.6±7.4
	FII	69.0±17.5 ^{b}		47.7±12.9 ^{b}	
	FIII	34.9±11.3 ^{b}		38.8±5.5 ^{b}	
	FIV		29.9±4.3		23.0±0.5
Avgoustiatis	TL	5.6±10.7 ^{a}		9.9±3.4 ^{a}	
	FI		22.7±11.1		7.6±3.1
	FII	43.6±6.6 ^{a}		22.9±4.9 ^{a}	
	FIII	10.3±4.7 ^{a}		37.8±3.5 ^{a}	
	FIV		33.5±8.0		22.3±7.3
Red thiako	TL	23.1±3.0 ^{a}		37.9±3.4 ^{a}	
	FI		14.6±16.8		15.4±6.0
	FII	91.5±3.4 ^{b}		92.1±10.3 ^{b}	
	FIII	10,2±9.7		12,0±7.7	
	FIV		9.5±0.5		9.6±0.4
Mavrodaphne of Kefalonia	TL	29.2±5.9 ^{a}		20,6±12.2 ^{a}	
	FI		11.9±7.0		0,0±3.6
	FII	36.4±28.1 ^{a}		36.5±26.0 ^{a}	
	FIII	0,0±9.9 ^{a}		31.6±5.5 ^{a}	

FIV	0,0±6.5	0,0±3.5

Within each wine different letters indicate significant differences between extracts based on one way ANOVA. A: activation

Supplementary Figure

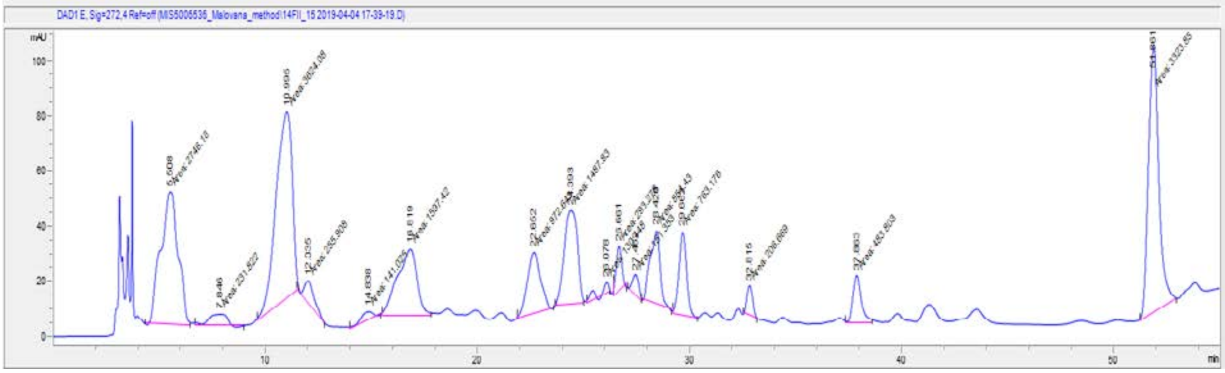

(a)

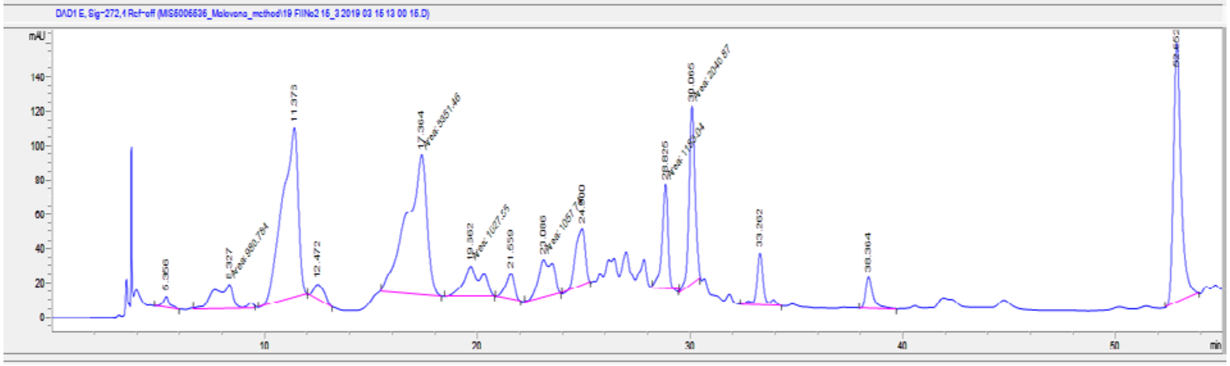

(b)

Supplementary Figure 1: Representative chromatographic HPLC separation of FII fraction from (a) one white (Robola of Kefalonia) and (b) one red (Vertzami) wine. Detection at 272 nm
